# Symbiotic Interaction Enhances the Recovery of Endangered Tree Species in the Fragmented Maulino Forest

**DOI:** 10.3389/fpls.2021.663017

**Published:** 2021-04-15

**Authors:** Cristian Torres-Díaz, Moisés A. Valladares, Ian S. Acuña-Rodríguez, Gabriel I. Ballesteros, Andrea Barrera, Cristian Atala, Marco A. Molina-Montenegro

**Affiliations:** ^1^Grupo de Biodiversidad y Cambio Global (BCG), Departamento de Ciencias Básicas, Universidad del Bío-Bío, Chillán, Chile; ^2^Instituto de Ciencias Biológicas, Universidad de Talca, Talca, Chile; ^3^Núcleo Científico Multidisciplinario, Universidad de Talca, Talca, Chile; ^4^Facultad de Ciencias, Instituto de Biología, Pontificia Universidad Católica de Valparaíso, Valparaíso, Chile; ^5^Facultad de Ciencias del Mar, Centro de Estudios Avanzados en Zonas Áridas (CEAZA), Universidad Católica del Norte, Coquimbo, Chile; ^6^Centro de Investigación en Estudios Avanzados del Maule (CIEAM), Universidad Católica del Maule, Talca, Chile

**Keywords:** *Nothofagus* spp., ruil, hualo, endangered tree species, restoration, Antarctica, fungal endophytes, functional symbiosis

## Abstract

Beneficial plant-associated microorganisms, such as fungal endophytes, are key partners that normally improve plant survival under different environmental stresses. It has been shown that microorganisms from extreme environments, like those associated with the roots of Antarctica plants, can be good partners to increase the performance of crop plants and to restore endangered native plants. *Nothofagus alessandrii* and *N. glauca*, are among the most endangered species of Chile, restricted to a narrow and/or limited distributional range associated mainly to the Maulino forest in Chile. Here we evaluated the effect of the inoculation with a fungal consortium of root endophytes isolated from the Antarctic host plant *Colobanthus quitensis* on the ecophysiological performance [photosynthesis, water use efficiency (WUE), and growth] of both endangered tree species. We also, tested how Antarctic root-fungal endophytes could affect the potential distribution of *N. alessandrii* through niche modeling. Additionally, we conducted a transplant experiment recording plant survival on 2 years in order to validate the model. Lastly, to evaluate if inoculation with Antarctic endophytes has negative impacts on native soil microorganisms, we compared the biodiversity of fungi and bacterial in the rhizospheric soil of transplanted individuals of *N. alessandrii* inoculated and non-inoculated with fungal endophytes. We found that inoculation with root-endophytes produced significant increases in *N. alessandrii* and *N. glauca* photosynthetic rates, water use efficiencies and cumulative growth. In *N. alessandrii*, seedling survival was significantly greater on inoculated plants compared with non-inoculated individuals. For this species, a spatial distribution modeling revealed that, inoculation with root-fungal endophytes could potentially increase the current distributional range by almost threefold. Inoculation with root-fungal endophytes, did not reduce native rhizospheric microbiome diversity. Our results suggest that the studied consortium of Antarctic root-fungal endophytes improve the ecophysiological performance as well as the survival of inoculated trees and can be used as a biotechnological tool for the restoration of endangered tree species.

## Introduction

Destruction and degradation of natural ecosystems by human actions are among the main causes of declines in global biodiversity (Saunders et al., 1991; Rands et al., 2010). Ecological restoration aims to recreate or accelerate the recovery of damaged or destroyed ecosystems due to these anthropogenic activities (Urbanska et al., 1997). Restoration is an expensive and time-consuming task which outcome can drastically differ, ranging from near-total success to total failure (Suding, 2011). Growing and planting seedlings is the more common, but also one of the most expensive strategies to restore plant populations (Zahawi and Holl, 2014). Hence, new methods that improve seedling establishment are crucial to perform successful restoration programs.

Although plants are generally considered as autonomous organisms, their ability to tolerate biotic and abiotic stress is highly dependent on functional symbiosis with beneficial microorganisms (Rodriguez and Redman, 2008; Smith and Read, 2008). Many studies have shown that fungi (e.g., arbuscular mycorrhizal fungi, fungal endophytes) and bacteria (e.g., plant growth promoting rhizobacteria) normally play an important role for plant development, growth, overall fitness, adaptability, and environmental stress tolerance (Saikkonen et al., 1998; Hardoim et al., 2015; Acuña-Rodríguez et al., 2020). These types of microbial partners have been extensively used as a biotechnological tool to increase the yield of crop species (reviewed in De Zelicourt et al., 2013; Acuña-Rodríguez et al., 2019). Recently, however, there is a growing interest for their use in ecological restoration (Asmelash et al., 2016). For instance, arbuscular mycorrhizal fungi have been used to restore degraded grasslands of central Asia (Zhang et al., 2012), Belgium (Torrez et al., 2016) and North America (Koziol and Bever, 2017). However, root-associated fungal endophytes have been seldom used in restoration ecology of woody species.

Interestingly, some studies (e.g., Brundrett et al., 1996; Petrini, 1996; Newsham et al., 2009; Ramos et al., 2018; Hereme et al., 2020) have suggested that positive symbiotic associations between plants and extremophile microbial symbionts could be a key factor for the adaptation and survival of plants in stressful environments (see Acuña-Rodríguez et al., 2020). Consistently, Rodriguez et al. (2008) showed that habitat-specific fungal endophytes, from extreme environments, confer stress tolerance to host plants from more benign environments. The Antarctic continent is among the harshest environments on Earth for plant life (Convey et al., 2008, 2014; Pointing et al., 2015), with plant establishment and survival limited by conditions such as low temperatures, desiccation, wind abrasion, photo-inhibitory radiation, and low availabilities of water and nutrients (Alberdi et al., 2002; Robinson et al., 2003; Wasley et al., 2006). Only two vascular plants have naturally colonized the Antarctic ecosystems; the Poaceae *Deschampsia antarctica* and the Caryophyllaceae *Colobanthus quitensis* (Moore, 1970; Biersma et al., 2020). Previous studies have shown that Antarctic root fungal endophytes improve the ecophysiological performance, growth, biomass, reproductive effort and/or survival of Antarctic plants (e.g., Torres-Díaz et al., 2016), but also benefit other xerophytic woody plant species from arid environments from the north of Chile (30°S) (Fardella et al., 2014) and crop species (Molina-Montenegro et al., 2016b, 2020). This suggests that the beneficial role of Antarctic fungal endophytes is not species-specific, and they could be also used as a potential biotechnological tool in restoration programs in phylogenetically unrelated plant species.

**FIGURE 1:**
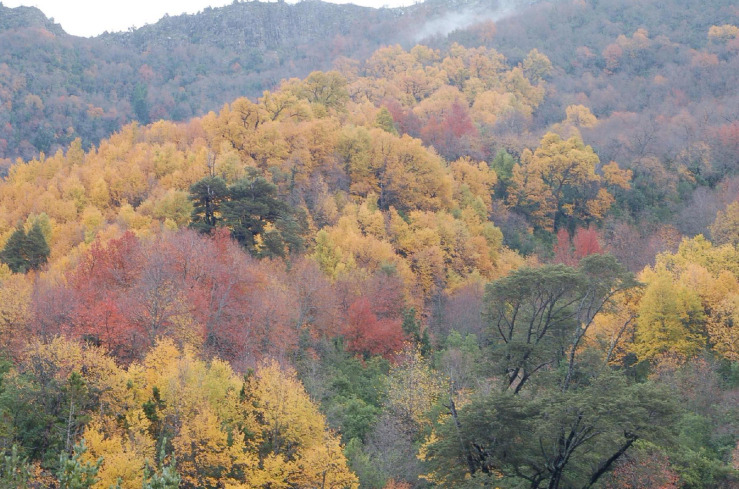
Landscape of the Maulino forest during austral autumn. Photographed by Claudio C. Ramírez.

*Nothofagus alessandrii* and *Nothofagus glauca* (Nothofagaceae), commonly known as “ruil” and “hualo,” respectively, are the dominant trees of the Maulino forests (Figure 1). While the restricted *N. alessandrii* is listed as endangered by the International Union for Conservation of Nature (IUCN), the more widely distributed *N. glauca* is listed as vulnerable (Benoit, 1989; Le-Quesne and Sandoval, 2001; IUCN, 2018). Simulations of the successional dynamic of this forest predict a drastic decline of sclerophyllous species and a growing dominance of commercial plantations (Bustamante et al., 2005). This reduction of the relative abundance of *N. glauca* may be the consequence, to some extent, of alterations in its process of establishment (pre-dispersal and/or pre-germination seed predation) as well as other anthropic factors such as habitat fragmentation (Burgos et al., 2008). On the other hand, *N. alessandrii* is one of the most primitive species within this genus and has been considered as a “living fossil” (*sensu* Van Steenis, 1972). This species has been historically restricted to an extremely narrow latitudinal range (∼100 km, 35–36°S) within the coastal Maulino forest in Chile. The forests dominated by these species have been increasingly destroyed and fragmented from the end of nineteenth century due to replacement with commercial plantations of *Pinus radiata* and *Eucalyptus* spp., and most of the stands represent secondary re-sprouts from stumps. Together with anthropogenic habitat fragmentation the species is currently threatened by anthropogenic fires. For instance, almost 50% of the stands dominated by ruil (339 hectares) were burnt due to a major fire that consumed nearly 14,000 ha in the Maule region during the 2016/17 austral summer. To date, the remaining 339 ha of ruil forest are composed by 186 stands, 95% of which are smaller than 2 ha. Hence, this species was included in 1998 in the “red list” of the IUCN (Barstow et al., 2020). On the base of its biological importance and its high vulnerability, this species was declared as a “natural monument” by the Chilean government in 2007. Currently, only 12% (0.42 km^2^) of its distributional range is under protection in the National System of Protected Areas (SNASPE) of Chile (Bustamante and Castor, 1998) in the “Los Ruiles” National Reserve. The reproduction and regeneration of this species under natural or controlled conditions is very limited and highly variable between populations (San Martín and Donoso, 1996; Olivares et al., 2005). For instance, in a restoration attempt only 90 out of 3,500 plants (2.6%) survived in the field (Antonio Cabrera, Universidad Católica del Maule, public interview published in http://www.lignum.cl, 2015). Thus, forestry managers normally find serious difficulties to repopulate this species, especially due to its high mortality at early plant developmental stages, for which new strategies are urgently needed to improve its survival and growth.

In this study we evaluated if the induced symbiosis between two species of root fungal endophytes, isolated from the Antarctic host plant *C. quitensis*, and the endangered trees *Nothofagus alessandrii* and *N. glauca*, could be used as a strategy to improve the physiological performance and growth of these endemic species. Similar beneficial effects of microbial partners such as arbuscular mycorrhizal fungi on plant establishment and survival have been also systematically evaluated in other systems (e.g., Grime et al., 1987; Koziol et al., 2018; Policelli et al., 2020). We specifically evaluated the effect of inoculation with a mix of Antarctic root fungal endophytes on the photosynthesis rate, water use efficiency (WUE) and relative growth of both tree species in greenhouse conditions. In addition, considering the restricted distribution and endangered status of *N. alessandrii*, we modeled how inoculation with this consortium could affect their spatial distribution through an ecological niche modeling approach based on the abiotic requirements of temperature and precipitation. We also compared seedling survival of inoculated (E+) and uninoculated (E–) seedlings of *N. alessandrii* in the field to validate our spatial distribution model. Finally, we performed 16s and ITS amplicon sequencing to study if the inoculation with Antarctic root fungal endophytes had an effect on the rhizospheric microbiome profiles associated with *N. alessandrii.*

## Materials and Methods

### Studied Plant Species

*Nothofagus alessandrii* is a long-lived broad-leaf caducifolious tree, characterized by anemophilous pollination and anemochorous seed dispersal. *N. glauca* is a caducifolious tree characterized by an outer bark that is thick and rough, with a markedly papery structure, and ashgray color. These species are endemic to the highly fragmented Maulino forest, which possesses a mesic-type climate, dominated by the narrowly distributed *N. alessandrii* and the slightly more widespread *N. glauca* (San Martín and Donoso, 1996) (Figure 1).

### Isolation of Antarctic Root-Fungal Endophytes and Inoculation Treatment

During the Antarctic expedition (ECA–52) organized by the Chilean Antarctic Institute (2015/16 austral summer), we collected 30 adult plants of *Colobanthus quitensis* close to the Polish Antarctic research station “Henryk Arctowski,” King George Island, South Shetland Islands, Antarctica (62°09′S). To obtain root-fungal endophytes we sterilized 1 cm fresh root segments of *C. quitensis* individuals with 70% ethanol for 1 min, followed by 3 min in 2% sodium hypochlorite and a final washing steps in distilled water for 2 min. Root segments were cultured for 10 days in Petri dishes with PDA medium with cloranfenicol (100 ml/L) at 18°C. Hyphae growing out of the root segments were re-inoculated in new plates with fresh medium. Different hyphae growing in the same root fragment that showed similar colony morphology were clustered. The isolates were maintained by routine sub-culturing with single-spore isolations. Finally, individual colonies formed were stored at 4°C until its utilization in both greenhouse and field experiments.

The fungal mix used for the inoculation was generated with the two most-abundant root fungal endophytes. These two strains were originally identified as *Penicillium chrysogenum* (Internal code HA) and *Penicillium brevicompactum* (Internal code HB) (see Molina-Montenegro et al., 2016b; Torres-Díaz et al., 2016) based on partial ITS (ITS1, ITS2, and the intercalary 5.8S rRNA gene) and LSU (the 28S nuclear ribosomal large subunit rRNA) genes.To achieve a better identification of these strains, in this study, we re-sequenced the internal transcriber spacer rDNA (ITS), but also sequenced two additional genes, β-tubulin (BenA) and Calmodulin (CaM), to achieve a more accurate identification of those fungal strains. Based on phylogenetic analyses, the two strains were re-assigned to *Penicillium rubens* (HA) and *Penicillium bialowienzense* (HB), respectively (see Supplementary Material 1). Despite this reassignment, this finding was consistent with the previous assignment as both species are grouped within the same taxonomic sections within the genus *Penicillium* (*Brevicompacta* and *Chrysogena*; see Supplementary Material 1).

To assess whether the mix of Antarctic root fungal endophytes (hereafter fungal endophytes) enhance the performance and restoration success we performed two manipulative experiments (greenhouse and field) and a spatial distribution modeling with (E+) and without (E–) fungal endophytes. First, we conducted a greenhouse experiment to evaluate the ecophysiological responses of *N. alessandrii* and *N. glauca* seedlings to inoculation with the fungal endophytes. Secondly, we conducted a transplant experiment in the field to evaluate survival of *N. alessandrii* seedlings. For both experiments, we selected 3-months-old seedlings obtained from “Reserva Los Ruiles” national reserve. Half of individuals were irrigated with an inoculum solution containing the mix of fungal endophytes. The solution consisted of a suspension of spores adjusted to 100 ml containing Tween-80 (0.05%), filtered through four layers of sterile cotton cheesecloth gauze. The spore concentration was adjusted to 10^–7^–10^–8^ spores/ml (50:50 from each species). Control plants did not receive the inoculum solution but were irrigated with tween-80 solution (0.05%) without fungal endophytes spores. Inoculated (E+) and non-inoculated (E–) seedlings of *N. alessandrii* were maintained in growth chambers at 22° C, a photon flux density (PFD) of 460 μmol m^–2^ s^–1^ and 14/10 h light/dark photoperiod during 15 days before assignment to different experiments and treatments. All seedlings were grown on native soil collected from Reserva Los Ruiles. Before the beginning of each experiment (greenhouse and field), the success of fungal infection was verified using light microscopy techniques and culture-based methods (Bacon and White, 2000).

### Ecophysiological Responses

The effect of experimental inoculation with the mix of Antarctic fungal endophytes was assessed on three ecophysiological response variables: net photosynthesis (A), instantaneous water use efficiency (WUE) and relative growth. A total of 60 seedlings of *N. alessandrii* and 120 seedlings of *N. glauca* were selected for these measurements, being the half of them assigned to +E treatment and the other half assigned to –E treatment. The net photosynthesis was measured on visually healthy leaves. Measurements were made on the same individual at 0, 15, 30, 60, and 90 days with an infrared gas analyzer (IRGA, Infra-Red Gas Analyzer, CIRAS-2, PP-Systems Haverhill, United States). We used gas exchange measurements to estimate the instantaneous WUE for photosynthesis as the ratio between photosynthetic rate (A) and transpiration rate (E), i.e., A/E ratio. This parameter has been widely used an estimator of plant water stress under different microclimatic conditions because increases in WUE are usually induced by a reduction in water availability (Lambers et al., 2008). The relative growth of seedling was measured at the nearest mm using a digital caliper at different times (0, 30, 60, and 90 days). This variable was evaluated comparing the difference in height at the end minus the height at the beginning of experimental period.

### Niche Modeling

The current probability of occurrence of *N. alessandrii* was modeled using eight WorldClim raster layers of bioclimatic variables^1^ related to temperature [mean daily temperature range (BIO2), temperature seasonality index (BIO4), maximal temperature of warmer month (BIO5), and minimal temperature of colder month (BIO6)], and water availability [annual precipitation (BIO12), seasonality of precipitations index (BIO15), precipitation of the warmer trimester (BIO18), and precipitation of the colder trimester (BIO19)]. These variables are the average for the 1970–2000 period and were considered at 30 arc-seconds resolution (∼1 km^2^) (Fick and Hijmans, 2017). The spatial points of reference for the modeling procedure were obtained from the coordinates of 15 fragments of *N. alessandrii* reported by Santelices et al. (2012).

To simulate the potential spatial distribution of *N. alessandrii* taking into account the effect of the Antarctic fungal endophytes, we considered the magnitude of the increase in WUE with respect to non-inoculated seedlings (14.7%, see Results) as a proxy of tolerance to lower water availability. To translate this physiological enhancement into the bioclimatic model, we reduced the data from those raster layers containing information about two precipitation variables (BIO12 and BIO19) in 14.7%. In this model we reduced these two variables maintaining all the rest constant, mimicking a 14.7% lower water requirement for *N. alessandrii*. Thus, we modeled the projected spatial distribution for *N. alessandrii* using the eight reported bioclimatic variables to extract the climatic raster data with true presences for plants without (E–: unmodified variables) with Antarctic fungal endophytes added (E+: BIO12 and BIO19 reduced). To evaluate the performance of our model (i.e., training and test) 100 random background points were extracted from the eight original raster layers. Raster WorldClim data was considered only for those cells within the Chilean territory between of 34.5°–36.5° S and 71°–73° W. A raster layer for the probability of occurrence of *N. alessandrii* in both scenarios was obtained using the R-package *dismo* (Hijmans et al., 2011). The mean values of probability where then calculated averaging 100 different iterations of the random forest (RF) algorithm that defines each raster cell probability in the *random Forest* R-package (Liaw and Wiener, 2002). In each run the coordinates of the 15 reported locations for *N. alessandrii* and 100 background points were randomly divided into two groups for the validation of the model (training: 70%; test: 30%). To estimate the final projected area in both E+ and E– scenarios we only retained those cells with a probability of occurrence ≥ 95%.

### Seedling Survival

In order to validate the results of our spatial distribution modeling, we conducted a transplant experiment in the field. A total of 100 seedlings of *N. alessandrii* were used for this experiment, where half of them (*n* = 50) were randomly assigned to inoculated with Antarctic fungal endophytes treatment (E+), and the other half (*n* = 50), to without Antarctic fungal endophytes or control treatment (E–). All seedlings were 3 months old and were transplanted in pairs (E+ and E–) with individual plants separated by 1 m. All seedling pairs were randomly transplanted within a 1km^2^ area and were separated by 10 m. The environmental conditions recorded in the field were typically Mediterranean, with a mean annual temperature of 22°C and a maximum of 36°C in January (Austral summer). Mean photosynthetic active radiation was 1078 μmol m^–2^s^–1^ with a maximum of 1523 in January. Soil water potential varies markedly throughout the year, ranging from –11.5 kPa in July to –39.1 kPa in February. The planting area is located 5 km away to the north from the current northern limit of the species distribution (35°03′3S; 72°04′4W) based on Santelices et al. (2012). In addition, the planting area considered for transplant is under the potential distributional range based in the spatial distribution modeling indicated above. Survival was recorded on each pair of seedlings (E+ and E–) in the field at 0, 15, 30, 60, 90, 180, 342, 410, and 733 days.

### Soil Sampling, DNA Extraction and Illumina Amplicon-Based Sequencing

To assess the effect of experimental inoculation with the mix of Antarctic fungal endophytes on rhizospheric soil microbial communities (in terms of α-diversity, species richness, evenness and dominance), we performed Amplicon sequencing of 16S and ITS DNA regions from a total of 15 rhizospheric soil samples. Ten samples, five (E+) and five (E–), were randomly selected from ecophysiological experiments. Since those seedlings were subjected to greenhouse conditions, which have the potential to affect soil microorganisms, we also included a third treatment consisting five “unmanipulated” soil samples (Control) taken from Reserva Nacional Los Ruiles and estimated their microbial diversity. From bulk soil samples, taken from the first 5 cm depth, we isolated rhizosphere soil by carefully detaching soil adhered to roots using a brush and tweezes. For all samples, DNA was extracted from an average of 0.25 g of soil (root-free) using the MoBio Power Soil DNA extraction kit following manufacturer’s instructions, resulting in an average of 0.24 μg of DNA per gram of soil. Libraries for sequencing were prepared by Macrogen Inc. (Seoul, South Korea). Template specific primers used in this study are universal primers targeting both 16s rRNA bacterial gene (341F/805R primers) and the fungal interspace transcribed spacer (ITS3/ITS4 primers), which are frequently used in bacterial and fungal metabarcoding studies (Herlemann et al., 2011; Op de Beeck et al., 2014).

### Analysis of the Soil Microbial Community

To identify bacteria and fungi present in the samples, metagenomic sequences needed to be filtered for quality, aligned into contigs and compared to existing sequence databases (Gawande et al., 2019). Sequences were run through Trimmomatic 0.36 (Bolger et al., 2014) to remove low-quality base pairs, sequencing adapters and reads shorter than 100 bp; the following parameters were used: SLIDINGWINDOW 4:15 MINLEN: 36. After trimming, microbiomes were analyzed using QIIME2 frame work (Bolyen et al., 2019). Trimmed sequences were quality filtered, and chimeras removed using DADA2 (Callahan et al., 2016). Taxonomic analysis was performed for bacterial libraries using a pre-trained Silva 132 99% OTUs based naïve Bayes classifier, while for fungi, the UNITE QIIME release for Fungi was used (UNITE Community, 2019). Taxonomic summary tables were generated for phylum-level abundance analysis by comparison between conditions with multiple *t*-tests using the Bonferroni correction method for false discovery rate. A principal coordinates analysis (PCoA) was performed to compare overall composition of communities within samples, based on Bray-Curtis dissimilarity index and EMPeror plugin (Supplementary Material 2; Vázquez-Baeza et al., 2013). In order to evaluate the potential impacts of inoculation with Antarctic endophytes on rhizosphere soil microbial community structure, we estimated and compared eight community indexes among treatments for bacteria and fungi. Considering their relevance for plant growth, we also compared ectomycorrhizae (hereafter, ECM) diversity among experimental treatments (E+, E–, and C). We specifically compared Shannon diversity index (H), Species Richness (S), Dominance (D), Evenness (E) and two abundance-based estimators of species richness indexes, Chao1 and ACE. All sequences (Fastq format) were deposited at NCBI^2^ under the BioProject code: PRJNA706688. Representative OTU sequences and taxonomic assignments were deposited in separated datasets at figshare platform for bacteria, fungi and ECM together with alpha diversity analysis^3^.

### Statistical Analyses

To evaluate the effect of endophyte inoculation of *N. alessandrii* and *N. glauca* seedlings on those variables measured along time (photosynthesis, WUE and cumulative growth), we used repeated measures (rm) ANOVAs taking the individual nested in time as the random error structure. ANOVA’s, *a posteriori* differences between treatments were evaluated using Honest Significant Differences (HSD) Tukey tests. Normality and homogeneity of variance were assessed with Shapiro-Wilks and Barlett tests, respectively (Sokal and Rohlf, 2012). Model fitting was performed with the *lme* function on the *nlme* R-package (Pinheiro et al., 2017). *A posteriori* comparison for the mixed models between experimental groups were performed by the comparison of their Estimated Marginal Means (EMMs) by factor levels as supported by the function pairs in the *emmeans* R-package (Lenth et al., 2018). Using data from *N. alessandri* E+ and E– seedlings surveyed in the field we obtained the Kaplan-Meier estimate of survival using the survfit function from the *survival* R-package (Therneau, 2015). To further compare the survival probabilities between experimental groups, for each species a Mantel-Haenszel log test was performed between the respective survival hazard ratios of inoculated (E+) and non-inoculated seedlings (E−), as allowed by the survdiff function on the same R-package. To evaluate the potential impacts of inoculation with Antarctic endophytes on the structure of microbial communities (bacteria, fungi and ECM), we compared Shannon (H), Species richness (S), Dominance (D), Evenness (E), ACE-1 and CHAO indexes using one-way ANOVA’s and Tukey’s HSD test for *post-hoc* comparisons. For those response variables that did not meet ANOVA assumptions (normality and homogeneity of variance) we used Kruskal-Wallis ANOVA’s and paired Mann-Whitney (U) test were used for *post-hoc* comparison.

## Results

### Ecophysiological Responses

**FIGURE 2:**
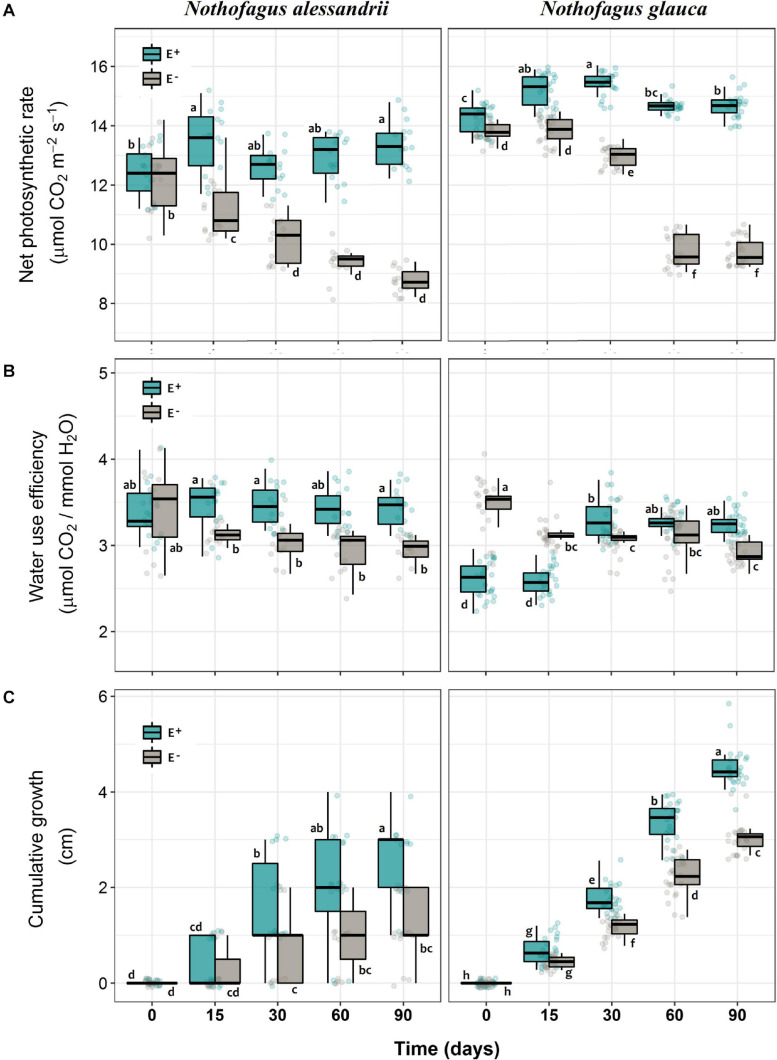
Photosynthetic responses **(A)**, water use efficiency **(B)**, and relative growth **(C)** of *N. alessandrii* and *N. glauca* seedlings inoculated (E+, calypso boxes) and not-inoculated (E–, gray boxes) with Antarctic root-fungal endophytes at different times. Box-plots indicate means and bars ± SD. Significant differences (Tukey HSD tests; α = 0.05) are highlighted with different letters.

Net photosynthesis (A) was significantly higher in seedlings of both species inoculated with the mix of Antarctic fungal endophytes than in non-inoculated seedlings (34.6% for *N. alessandrii*; 50.9% for *N. glauca*) (Figure 2A and Table 1). The significant interaction between inoculation and time, also observed in both species (Table 1), indicates that the photosynthetic rate was stable through time for inoculated seedlings, but drastically reduced in non-inoculated ones (Figure 2A). Similarly, WUE was significantly higher in plants inoculated with Antarctic fungal endophytes than in non-inoculated individuals (14.7% *N. alessandrii*; 12.1% for *N. glauca*) (Figure 2B and Table 1). The interaction factor (Inoculation x Time) was also significant, indicating that inoculated seedlings are more efficient in their water-use through time (Table 1). However, while for the inoculated *N. alessandrii* seedlings this effect was stable through time, for *N. glauca* it was only observed after 90 days (Figure 2B). The mean cumulative growth of both species seedlings after 90 days was significantly higher in inoculated than in non-inoculated plants (47.2% *N. alessandrii*; 48.3% for *N. glauca*) (Figure 2C and Table 1). Consistently, the interaction factor (Inoculation x Time) revealed that inoculation with Antarctic fungal endophytes promoted seedling growth along time in both species compared to control plants (Figure 2C). Interestingly, despite both species showed enhanced growths, this effect was more pronounced in *N. glauca*, which shows a stepper increase and a consistent lower variability during the experiment.

**TABLE 1:** Repeated measures ANOVA’s results showing the effect of endophyte inoculation on the net photosynthetic rate (A max), water use efficiency (WUE) and cumulative growth in seedlings of two endangered Maulino forest trees: *Nothofagus alessandrii* and *Nothofagus glauca*.

**Species**	**Response Variable**	**Source of variation**	**df**	**SS**	**MS**	***F***	***p***
***N. alessandrii***	**Photosynthesis (*A*_max_)**	Inoculation	1	259.52	259.52	468.19	<0.0001
		Inoculation × Time	4	83.67	20.92	37.74	<0.0001
		Residuals	70	38.8	0.55		
	**WUE**	Inoculation	1	4.601	4.601	70.971	<0.0001
		Inoculation × Time	4	1.194	0.298	4.604	0.0023
		Residuals	70	4.538	0.065		
	**Cumulative growth**	Inoculation	1	15.36	15.36	40.523	<0.0001
		Inoculation × Time	4	9.107	2.277	6.006	0.0003
		Residuals	70	26.533	0.379		
***N. glauca***	**Photosynthesis (*A*_max_)**	Inoculation	1	502.1	502.1	2742.8	<0.0001
		Inoculation × Time	4	208.2	52	284.3	<0.0001
		Residuals	120	22	0.2		
	**WUE**	Inoculation	1	1.144	1.144	24.99	<0.0001
		Inoculation × Time	4	13.814	3.453	75.44	<0.0001
		Residuals	120	5.493	0.046		
	**Cumulative growth**	Inoculation	1	28.84	28.842	283.42	<0.0001
		Inoculation × Time	4	18.15	4.538	44.59	<0.0001
		Residuals	120	12.21	0.102		

*The overall differences by the experimental factor (Inoculation) and its efect on time (Inoculation x Time) are shown. Significant probability values (p < 0.05) are highlighted in red.*

On the other hand, in an additional experiment conducted with both species we showed that for the majority of these ecophysiological parameters as well as for survival, the inoculation with the mix of fungal endophytes, significantly improve these responses compared with non-inoculated individuals, but also compared with a commercial product based in a mix of mycorrhizas (Safer-Mycorrhizas M.A.), which has been demonstrated to improve the growth and abiotic tolerance in plants (Supplementary Material 3).

### Niche Modeling

**FIGURE 3:**
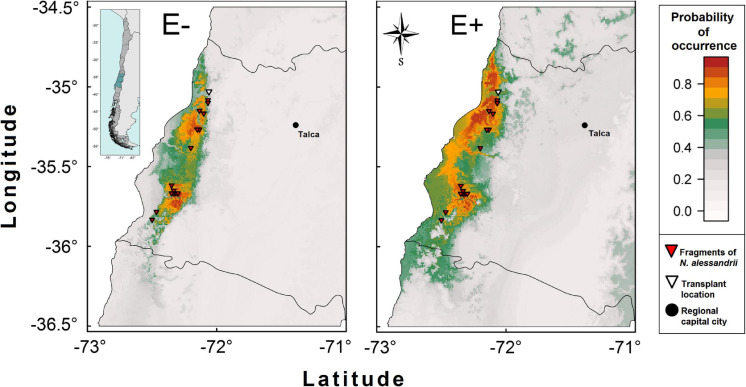
Spatial distribution projection for *N. alessandrii* without (E–) and with (E+) the Antarctic root-fungal endophytes. The E– scenario represents the current distribution of the species based on the localities with *N. alessandrii* reported by Santelices et al. (2012) and eight WorldClim bioclimatic variables related with precipitation and temperature. The functional effect of the root inoculation scenario E+ on the spatial distribution was modeled by reducing two (BIO12 and BIO19) out of the eight variables related to precipitation in 14.7%.

The modeling of distribution of *N. alessandrii* for the E– (unchanged) condition was highly correlated with the current distribution of the species. The estimated mean area considering cells with occurrence probabilities ≥ 95% (408.3 ± 58.5 ha) was similar to that reported for the species (339 ha by Santelices et al., 2012). This suggests that temperature and precipitation are good predictors of the distribution of the species (Figure 3). For the E+ condition (with a reduced water requirement of 14.7%), the potential area of distribution of *N. alessandrii* for a ≥ 95% of occurrence increased 2.8-times, from 408.3 ± 58.5 ha to 1175.8 ± 102.9 ha, being concentrated around current populations but also spreading into new areas, particularly northward (Figure 3).

### Seedling Survival

**FIGURE 4:**
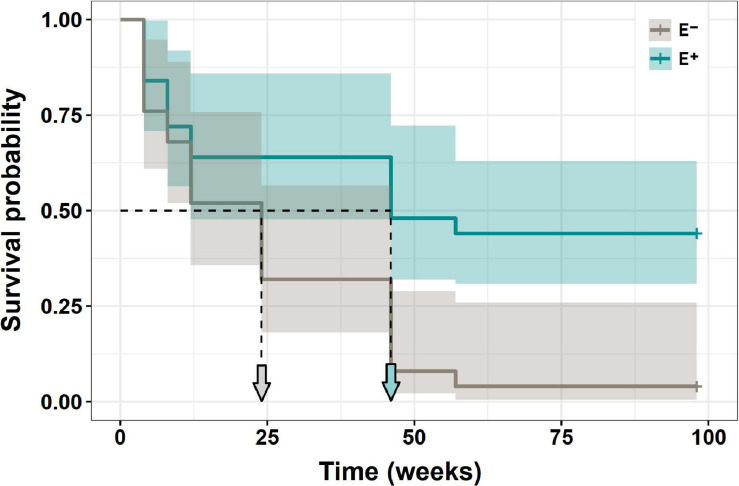
Survival of *N. alessandrii* seedlings with (E+, calypso line) and without (E–, gray line) Antarctic root fungal endophytes, along 2 years after field transplants.

The survival of *N. alessandrii* seedlings was significantly higher (Mantel-Haenszel test: χ^2^ = 8.7; df = 1; *p* = 0.0032) when inoculated with the mix of Antarctic fungal endophytes, compared with non-inoculated seedlings (Figure 4). After more than 2 years (733 days), all non-inoculated individuals died, while nearly 40% of the inoculated seedlings survived. While inoculated seedlings reached 50% mortality after ∼8–9 months, non-inoculated seedlings reached 50% mortality after ∼2–3 months (Figure 4).

### Metagenomics Sequencing of Rhizospheric Soils With (E+) or Without (E−) Endophytes

**FIGURE 5:**
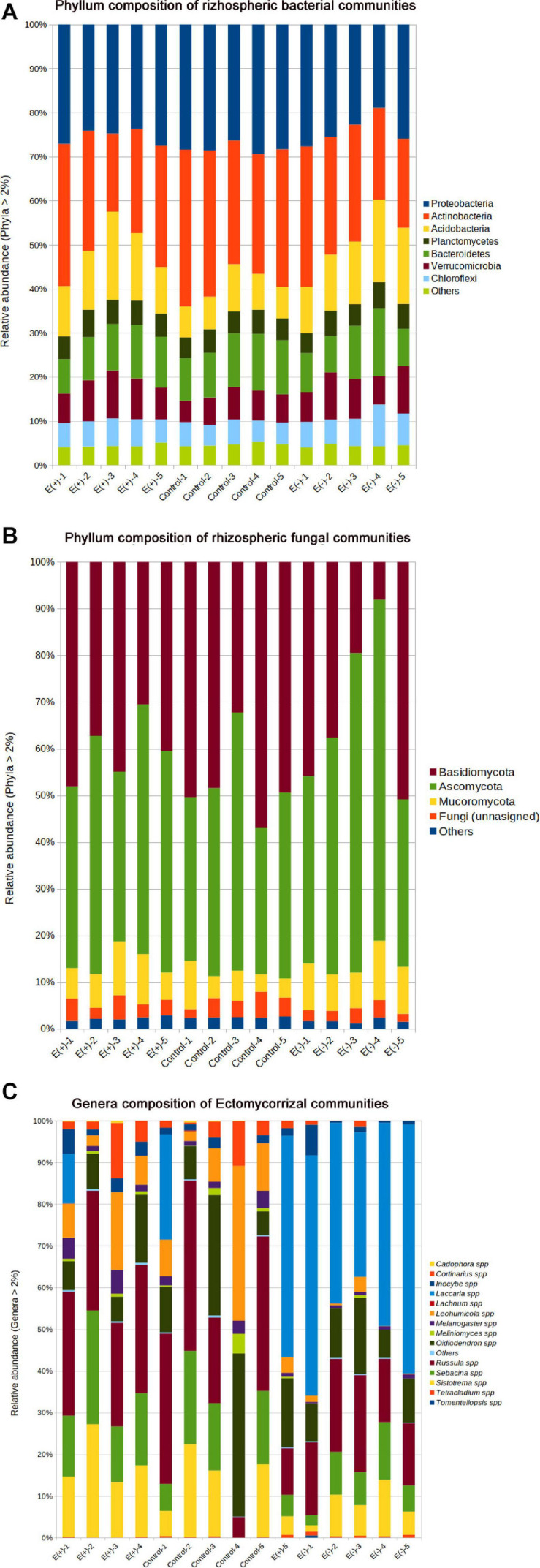
Relative abundance represented in rhizospheric soil samples associated to *Nothofagus alessandrii*. E+: with endophytes; Control: forest soil; E–: seedlings without endophytes. **(A)** Bacterial Phyla. **(B)** Fungal Phyla and **(C)** Ectomycorrizae genera.

A total of 41 bacterial phyla and 10 fungal phyla were detected from all rhizospheric soil samples analyzed in this study (Figures 5A,B). From the identifiable bacterial sequences, the majority belonged to phylum Actinobacteria, followed by Proteobacteria and Acidobacteria (Figure 5A). In the case of fungi, the most abundant phyla were Ascomycota and Basidiomycota (Figure 5B).

Globally, inoculation with Antarctic endophytes differences did not affect community structure of bacteria (Figure 5A and Table 2) and fungi (Figure 5B and Table 2). Nonetheless, we detected significant differences among treatments in the diversity (Shannon H index) of ectomycorrhizae. Specifically, ectomycorrhizae diversity of E+ and control soils was similar between them (*H-*index = 1.87 and *H-*index = 1.82, respectively) but higher than E– treatment (*H*-index = 1.46) (Figure 5C and Table 2). The lower species richness in E– soils was paralleled with increased dominance and reduced evenness in E+ soils, although those differences were not statistically significant (Figure 5C and Table 2). Detection of ectomycorrhiza fungi was accomplished by selecting all OTUs matching with ectomycorrhiza genera/species listed in NCBI Taxonomy database [ectomycorrhiza (Name Tokens)]. The most abundant genera/species were *Russula* spp., *Sistotrema* spp., *Laccaria* spp., and *Leohumicola minima* (Figure 5C).

**TABLE 2:** Summary of the results of community structure indexes comparisons among three experimental conditions (E+ = native soil inoculated with Antarctic endophytes, E– = native soil non-inoculated with Antarctic endophytes and Control = unmanipulated native soil) for Bacteria, Fungi and ECM (ectomycorrizae).

**Taxonomic group**	**Community parameter**	***d.f.***	**ANOVA**			**Kruskal-Wallis**		**Mean values**		
			***MS***	***F***	***P***	***H***	***P***	**E+**	**E–**	**Control**
Bacteria	Shannon (H)	2	–	–	–	3.12	0.210	7.09	6.87	6.88
	Species richness (S)	2	–	–	–	4.74	0.093	3963	3382	3542
	Dominance (D)	2	–	–	–	4.88	0.087	0.003	0.004	0.006
	Evenness (E)	2	0.001	0.37	0.692	–	–	0.307	0.304	0.279
	ACE	2	–	–	–	4.74	0.093	4030	3446	3608
	Chao1	2	–	–	–	4.74	0.093	4057	3471	3642
Fungi	H—Shannon	2	0.114	3.61	0.059	–	–	3.96	3.70	3.69
	S—Species richness	2	8495	0.99	0.396	–	–	520	443	456
	D—Dominance	2	3.809	3.81	0.052	–	–	0.045	0.059	0.060
	E—Evenness	2	0.001	0.33	0.725	–	–	0.103	0.097	0.090
	ACE	2	8509	1.09	0.396	–	–	520	443	456
	Chao1	2	8715	1.00	0.396	–	–	520	443	456
ECM	H—Shannon	2	0.250	4.39	0.037	–	–	1.87^a^	1.46^b^	1.82^a^
	S—Species richness	2	10.87	0.25	0.708	–	–	29.8	27.0	27.6
	D—Dominance	2	0.018	3.16	0.078	–	–	0.241	0349	0.247
	E—Evenness	2	–	–	–	3.84	0.146	0.224	0.169	0.255
	ACE	2	10.87	0.25	0.708	–	–	29.8	27.0	27.6
	Chao1	2	10.87	0.25	0.708	–	–	29.8	27.0	27.6

*Community parameters were compared with one-way ANOVAs and Tukey tests when assumptions of normality and homogeneity of variance were meet. Differences among treatments were addressed with Kruskal-Wallis (H) tests when the assumptions of ANOVA were not meet. Mean values of each community parameter for all treatment are also indicated. Significant probability values (p < 0.05) are highlighted in red. Different uppercase letters indicate differences between treatments.*

## Discussion

The results of our study provide conclusive evidence showing that the mix of Antarctic fungal endophytes (*P. rubens* and *P. bialowiezense*) has significant beneficial effects on the ecophysiological performance of the endangered endemic trees *Nothofagus alessandrii* and *N. glauca*, while not displaying any significant effects on the relative abundance and composition of the associated rhizospheric microbiomes. The positive effect of Antarctic fungal-endophytes seems to be ubiquitous as they are able to establish functional symbioses with plants species from a phylogenetically wide range of plant families such as Nothofagacae (this study), Caryophyllaceae, Poaceae, Asteraceae, Bromeliacae, and Fabacae (Fardella et al., 2014;, Molina-Montenegro et al., 2016a; Torres-Díaz et al., 2016).

On the other hand, spatial distribution modeling suggested that *N. alessandrii* could expand its distributional range northward if seedlings are inoculated with the mix of Antarctic fungal endophytes. The survival of inoculated seedlings measured in the field gives support to the predicted population expansion. Together, these results indicate that Antarctic fungal endophytes have the potential to be used as a biotechnological tool in restoration programs of the endangered *N. alessandrii* and *N. glauca*, and possibly other endangered tree species, with promising results even when are compared to commercial products containing other species of mycorrhizas.

The inoculation with the mix of Antarctic fungal endophytes improved the physiological status of both tree species seedlings through increases in net photosynthesis and WUE. Although morphological changes in the root system, which can be an efficient strategy to increase water uptake under drought, were not assessed, the greater relative growth and higher photosynthetic rate –at the end of the experiments– could be attributed to the positive effect of endophytes at the root–level. It has been suggested that endophyte–induced variations in the rhizosphere, such as production of sugars, proteins, and/or enzymes that avoid cell damage in membranes, allow some plants to cope with stressful environmental conditions that can be found in Mediterranean ecosystems (Baltruschat et al., 2008). Thus, endophyte inoculation of both tree species roots could be an efficient strategy to maintain high photosynthetic capacity as well as high WUE and, hence, a higher survival percentage in field. Consequently, Antarctic fungal endophytes could help to reduce the costs of irrigation in restoration initiatives since plants with higher WUE would require less water for the same carbon gain. Nonetheless, our findings should be viewed with caution. Nuñez et al. (2015) argued that unexpected outcomes such as promoting invasion of non-native plants or changing competitive relationships among native species could result from inoculation with non-native microorganisms. Nevertheless, in this study the manipulative inoculation did not affect bacteria and fungi soil microorganisms, indicating that the use of Antarctic fungal endophytes has not negative indirect effects microorganism inhabiting the rhizozpheric soil of the ruil individuals. However, interestingly, we found a significant reduction in the ECM community diversity in soils from non-inoculated plants (Table 2) probably to increased dominance of some genus such as *Lacaria* (Figure 5C). This finding could be explained by the environmental conditions E– seedlings were exposed to inside greenhouse, and by the reduced physiological status of E– seedlings that might promote the dominance of some ECM taxa, reducing the evenness. Nonetheless, further research is needed to confirm this potential explanation.

The spatial distribution modeling for *N. alessandrii* without endophytes was very close to the currently reported distribution of *N. alessandrii*. Interestingly, when the spatial distribution predicted for *N. alessandrii* was projected considering the increased WUE due to Antarctic fungal endophytes (14.7%), the range of distribution increased by threefold mainly northward. This result suggests that Antarctic fungal endophytes could be used to improve restoration success of the southern limit of *N. alessandrii*, but also in zones beyond its northern range limit. In a recent study, Alarcón and Cavieres (2015) performed a niche modeling to evaluate how different climate change scenarios, based on rainfall reductions and temperature increase expected for the next century, would affect the distribution of eight dominant trees from Maulino forest. Specifically, Alarcón and Cavieres (2015) estimated that *N. alessandrii* would suffer the worst reduction in habitat (42%) among the studied species. Our results suggest that Antarctic fungal endophytes may help mitigate the negative impacts of future global climate change on this species. However, this prediction should be experimentally tested by future field studies.

Climate change and anthropogenic land use changes are among the main threats to the health of Mediterranean-type forests and woodlands (Brouwers et al., 2013). One of the main aims of restoration ecology is to recreate or accelerate the recovery of an ecosystem that has been degraded, damaged or destroyed by human activities (Urbanska et al., 1997). One of the first steps required to assess the restoration of Mediterranean woodlands is to understand its generation dynamics (Vallejo et al., 2012). Unlike other widespread *Nothofagus* spp., such as *N. antarctica*, *N obliqua* or *N. pumilio* (Donoso, 1995; Pollmann and Veblen, 2004; Mathiasen and Premoli, 2013; Fajardo et al., 2016), the regeneration ecology of *N. alessandrii* and *N. glauca* remains poorly understood (but see Olivares et al., 2005). In these species, natural regeneration occurs after small-scale disturbance, such as tree-falls. This suggests that the optimal recruitment in *N. alessandrii* could be reached in forest gaps with intermediate levels of shade and moderate environmental stress (drought and light intensity). This is consistent with the results of Santelices et al. (2015), who found that *N. alessandrii* seedlings performed better under intermediate shade (41–50% PAR) than under full sunlight conditions (100% PAR). In a recent study, Loewe et al. (2017) experimentally evaluated the effects of shading and soil fertilization on the survival and stem length of *N. alessandrii* seedlings after one and two growing seasons under field conditions. These authors found that, after 1 year, shading and soil fertilization (nitrogen addition) increased seedling survival by 35 and 20%, respectively. Moreover, soil fertilization (600 mg L^–1^ of nitrogen) increased mean stem length from 10 to 50 cm. After 2 years, shading increased plant survival from 20 to 60%. These results indicate that *N. alessandrii* is highly sensitive to photo-inhibitory conditions of light and nutrient. Interestingly, we found that inoculation with Antarctic endophytes increased seedling survival from zero to ∼40% in the field, which is similar to the effect of nutrient addition found by Loewe et al. (2017). Nonetheless, our results should be viewed with caution and future field experiments should evaluate whether inoculation and soil fertilization plus shading might interact additively or synergistically in improving seedling growth and survival and, thus, long-term restoration success.

## Conclusion

The Mediterranean zone of central Chile (33–39°S) is claimed to be a biodiversity hotspot as a result of its high species richness and endemism (Myers et al., 2000). Nonetheless, this zone has been subjected to extensive land use change for agronomic and forestry uses (Echeverria et al., 2006). In addition to the high historical levels of habitat fragmentation experienced by *N. alessandrii*, and *N. glauca* in a lesser degree, in the recent years, the frequency and intensity of the anthropogenic fires has increased the risk of extinction of these emblematic species. Some estimates indicate that the last summer (February 2017), almost 50% of the stands dominated by *N. alessandri* (150 hectares) were burn by a major fire that consumed nearly 14,000 hectares in the Maule region. Our results indicate that Antarctic fungal endophytes could be used to restore or to “create” new stands in recently burnt areas where environmental stress is high due to drought. In addition to the threats mentioned above, global warming is another serious threat for these tree species as climate change models project drastic increases in temperature and reductions in precipitation for the Mediterranean-type climate zone of central Chile (CONAMA, 2007; Intergovernmental Panel on Climate Change, 2016). The results of the spatial distribution modeling and field survival experiment indirectly indicate that inoculation with Antarctic fungal endophytes could improve the drought tolerance of *N. alessandrii*, but without affecting plants’ rhizospheric microbiome. This could be useful to mitigate the negative impacts of the future climatic conditions expected by climate change models for the next century in the Mediterranean climate zone of central Chile. Thus, we call for further experimental research under the field conditions, such as warming experiments with open top chambers (OTCs), after wild-fires or simulated desertification, testing how Antarctic fungal endophytes could mitigate the negative impacts of climate change on these highly endangered endemic species and for field studies on the use of these endophytes in long-term restoration.

## Data Availability Statement

The datasets presented in this study can be found in online repositories. The names of the repository/repositories and accession number(s) can be found below: The raw data from the 16s and ITS libraries are in the NCBI Short Read Archive (BioProject PRJNA706688): Rhizospheric soil samples associated with N. alessandrii (Ruil)—Raw sequence reads. ITS sequence data and assembled 16s (Operational Taxonomic Units, OTUs) are available at: https://doi.org/10.6084/m9.figshare.c.5337266.v2 MM-M; CT-D; MV; IA-R; GB; AB; et al. (2021): Symbiotic interaction enhances the recovery of endangered tree species in the fragmented Maulino Forest. figshare. Collection. https://doi.org/10.6084/m9.figshare.c.5337266.v2.

## Author Contributions

MM-M, IA-R, and CT-D designed the experiments. MM-M, IA-R, AB, and CA performed the experiments. MM-M, GB, IA-R, MV, and CT-D analyzed the data. MM-M wrote the manuscript along with IA-R, CA, GB, and CT-D. All authors reviewed the manuscript.

## Conflict of Interest

The authors declare that the research was conducted in the absence of any commercial or financial relationships that could be construed as a potential conflict of interest.

## Footnotes

1 http://worldclim.org/version2

2 https://www.ncbi.nlm.nih.gov/

3 https://doi.org/10.6084/m9.figshare.c.5337266.v1
