# Supplementary material for: Symbiotic Interaction Enhances the Recovery of Endangered Tree Species in the Fragmented Maulino Forest
Source: Front Plant Sci. 2021 Apr 15;12:663017. doi: 10.3389/fpls.2021.663017 (PMC8081837; doi:10.3389/fpls.2021.663017)

**Supplementary material S1.** Methods and results associated to phylogenetic reidentification of the two Antarctic fungal strains used to restore two endangered Maulino forest trees.

**Molecular methods**

DNA extraction and PCR reactions were carried out following the protocol described in Torres-Díaz et al., 2016). Annealing temperatures were 52ºC for BenA and CaM, and 57ºC for ITS. For molecular identification we sequenced ﻿β-tubulin (*BenA*), calmodulin (*CaM*) and the ﻿internal transcribed spacer rDNA area (ITS). Forward and reverse strands of both isolates (HA and HB) were corrected for misreads and merged into one sequence file using CodonCode Aligner v3.6.1 (CodonCode Corporation, Dedham, MA, USA). The taxonomic affiliation was defined using a barcoding approach, and the species recognition was determined through phylogenetic reconstructions. For species molecular identification, *ITS* sequences of both isolates were compared against the *ITS* RefSeq for fungi database available in the National Centre for Biotechnological Information (NCBI) using the nucleotide BLAST program (Altschul et al., 1990). A Local BLAST analysis was implemented for *BenA* sequences and we used the ﻿GenBank accession numbers provided by Visagie et al. (2014) as reference database for accepted *Penicillium* species. For the phylogenetic reconstructions the sequences of both strains were included in a comprehensive dataset depicting main lineages of *Penicillium* (Table 1). Homologous DNA sequences were aligned independently for each locus using MUSCLE v3.8.31 (Edgar, 2004) in order to generate a multiple sequence alignment (MSA). ﻿Successively, each MSA was trimmed according to the reliability measure estimated using the ﻿Transitive Consistence Score (TCS) available in the T-Coffee web server (Chang, Di Tommaso & Notredame, 2014; Chang et al., 2015). Traditional phylogenetic reconstructions were performed under the algorithms of Maximum Likelihood (ML) and Bayesian Inference (IB). The best sequence evolution model for each locus was selected using bModelTest v1.2.1 (Bouckaert & Drummond, 2017). Later, the sequences were concatenated and posterior analyses were implemented using a partitioned dataset considering each locus (*BenA*, *CaM* and ITS). ML reconstruction was performed in RAxML v8.2.12 (Stamatakis, 2014) and the node support was obtained by performing a bootstrap analysis of 1000 pseudo-replicates. Bayesian reconstruction was performed in MrBayes3 v2.6.7 (Ronquist et al., 2012), using the Markov Chain Monte Carlo (MCMC) method. ﻿We performed four parallel runs with 20 million generations each to infer a maximum clade credibility tree using a burn-in period of 25%. ML and IB reconstructions were performed in the CIPRES cluster of the San Diego Supercomputer Center (Miller, Pfeiffer & Schwartz, 2010). The edited sequences for HA (*BenA*: MW702324, *CaM*: MW75145, ITS: MW725156) and HB (*BenA*: MW702325, ITS: MW725157) are available at NCBI website.

**Results**

While three loci were sequenced and used for phylogenetic reconstructions in HA (*ITS*, *BenA* and *CaM*), only two were available for HB (*ITS* and *BenA*). The summary of the best molecular evolution models obtained for each gene is shown in Table 1. The phylogenetic analyses (see Fig.1) revealed that the closer affinities to HA and HB fungal strains were *P. rubens* and *P. bialowienzense*, belonging to Chrysogena and Brevicompacta section, respectively. Those results were consistent with BLAST analyses for both species. For HA and HB, the highest similarities for ITS were with *P. rubens* (Score = 1051; Identity Percentage = 99.48%) and *P. bialowiezense* (Score = 911; Identity Percentage = 100%), respectively. Local BLAST indicated that the highest similarity for HA considering *BenA* was with *P. rubens* (GenBank: JF909949; Score = 850; Identity Percentage = 100%), followed by *P. chrysogenum* (GenBank: AY495981; Score = 882; Identity Percentage = 98%). In turn, *Ben A* sequence revealed that the highest similarity of HB was with *P. bialowiezense* (GenBank: AY674439; Score = 852; Identity Percentage = 97%).

**Table 1**. Summary of the evolutionary models obtained using bModelTest and nucleotide composition for each gene.

| **Marker** | **Size (pb)** | **Model** | **Nucleotide Composition** | | | |
| --- | --- | --- | --- | --- | --- | --- |
|  |  |  | **A** | **C** | **G** | **T** |
| *BenA* | 287 | TN93+I+G | 0.192 | 0.300 | 0.267 | 0.241 |
| *CaM* | 290 | HKY+I+G | 0.269 | 0.265 | 0.283 | 0.183 |
| *ITS* | 590 | TN93+I+G | 0.203 | 0.292 | 0.291 | 0.214 |
| Total | 1167 |  |  |  |  |  |

**References**

Altschul SF, Gish W, Miller W, Myers EW, Lipman DJ. 1990. Basic local alignment search tool. *Journal of Molecular Biology* 215:403–410. DOI: 10.1016/S0022-2836(05)80360-2.

Bouckaert RR, Drummond AJ. 2017. bModelTest: Bayesian phylogenetic site model averaging and model comparison. *BMC Evolutionary Biology* 17:42. DOI: 10.1186/s12862-017-0890-6.

Chang J-M, Di Tommaso P, Lefort V, Gascuel O, Notredame C. 2015. TCS: a web server for multiple sequence alignment evaluation and phylogenetic reconstruction: Figure 1. *Nucleic Acids Research* 43:W3–W6. DOI: 10.1093/nar/gkv310.

Chang J-M, Di Tommaso P, Notredame C. 2014. TCS: A New Multiple Sequence Alignment Reliability Measure to Estimate Alignment Accuracy and Improve Phylogenetic Tree Reconstruction. *Molecular Biology and Evolution* 31:1625–1637. DOI: 10.1093/molbev/msu117.

Edgar RC. 2004. MUSCLE: multiple sequence alignment with high accuracy and high throughput. *Nucleic Acids Research* 32:1792–1797. DOI: 10.1093/nar/gkh340.

Miller MA, Pfeiffer W, Schwartz T. 2010. Creating the CIPRES Science Gateway for Inference of Large Phylogenetic Trees. *Proceedings of the Gateway Computing Environments Workshop (GCE)* 1:1–8.

Ronquist F, Teslenko M, van der Mark P, Ayres DL, Darling A, Hohna S, Larget B, Liu L, Suchard MA, Huelsenbeck JP. 2012. MrBayes 3.2: Efficient Bayesian Phylogenetic Inference and Model Choice Across a Large Model Space. *Systematic Biology* 61:539–542. DOI: 10.1093/sysbio/sys029.

Stamatakis A. 2014. RAxML version 8: a tool for phylogenetic analysis and post-analysis of large phylogenies. *Bioinformatics (Oxford, England)* 30:1312–3. DOI: 10.1093/bioinformatics/btu033.

Torres-Díaz, C., Gallardo-Cerda, J., Lavin, P., et al. (2016). Biological Interactions and Simulated Climate Change Modulates the Ecophysiological Performance of *Colobanthus quitensis* in the Antarctic Ecosystem. PLoS One 11:e0164844. doi:10.1371/journal.pone.0164844

Visagie CM, Houbraken J, Frisvad JC, Hong S-B, Klaassen CHW, Perrone G, Seifert KA, Varga J, Yaguchi T, Samson RA. 2014. Identification and nomenclature of the genus Penicillium. *Studies in Mycology* 78:343–371. DOI: 10.1016/j.simyco.2014.09.001.

**Figure 1. Phylogenetic tree based on Bayesian inference and Maximum Likelihood (BI/ML)**. **For this tree, the final ML Optimization Likelihood was -15840.02.**


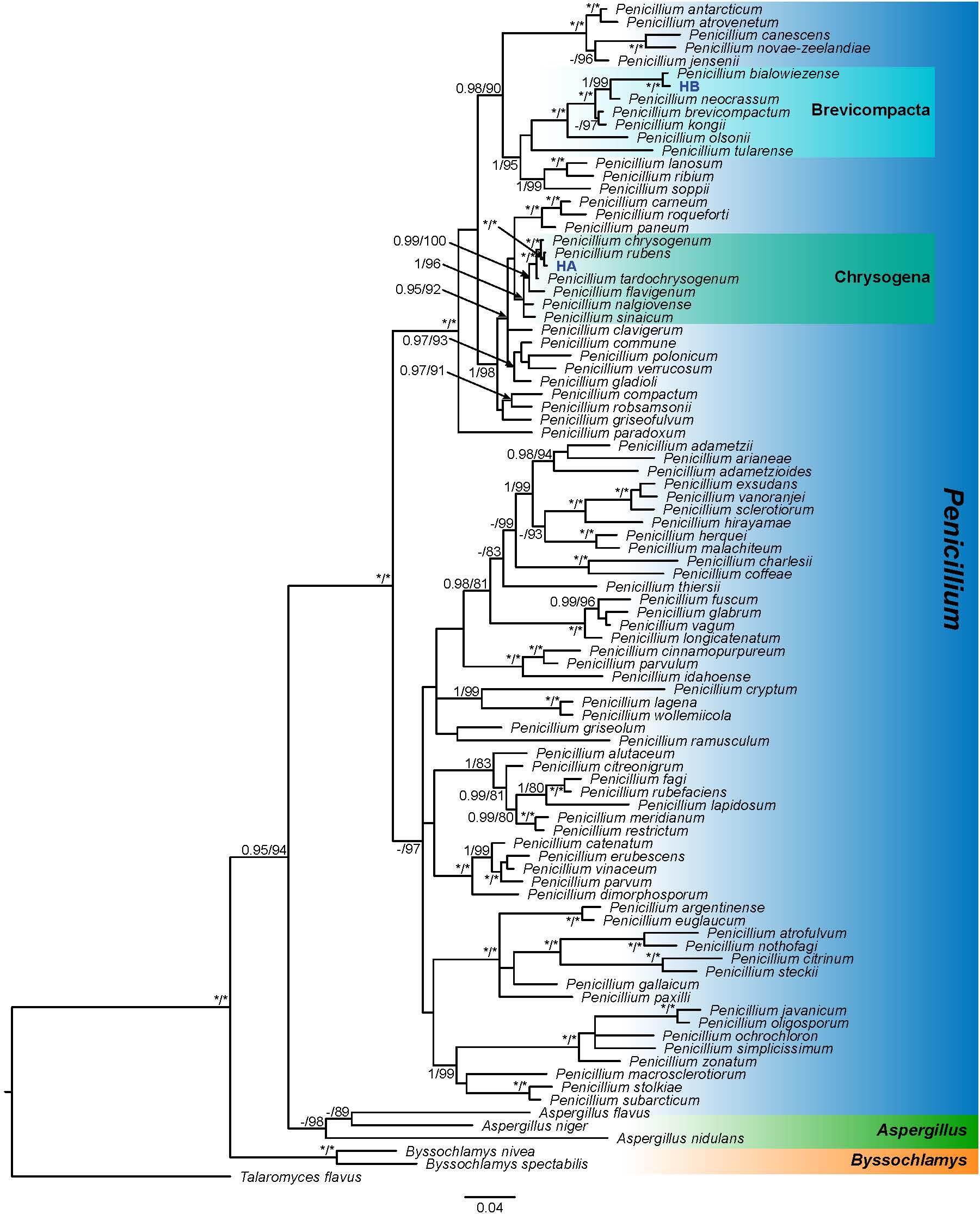

Supplement: Supplementary file 1 [file Data_Sheet_1.doc]
