# Supplementary material for: Symbiotic Interaction Enhances the Recovery of Endangered Tree Species in the Fragmented Maulino Forest
Source: Front Plant Sci. 2021 Apr 15;12:663017. doi: 10.3389/fpls.2021.663017 (PMC8081837; doi:10.3389/fpls.2021.663017)

**Supplementary material S2**.

X: Results of PCoA plot showing bacterial (A) and fungi (B) diversity based on Bray-Curtis dissimilarity, according to experimental treatments (E+, E– and Control).


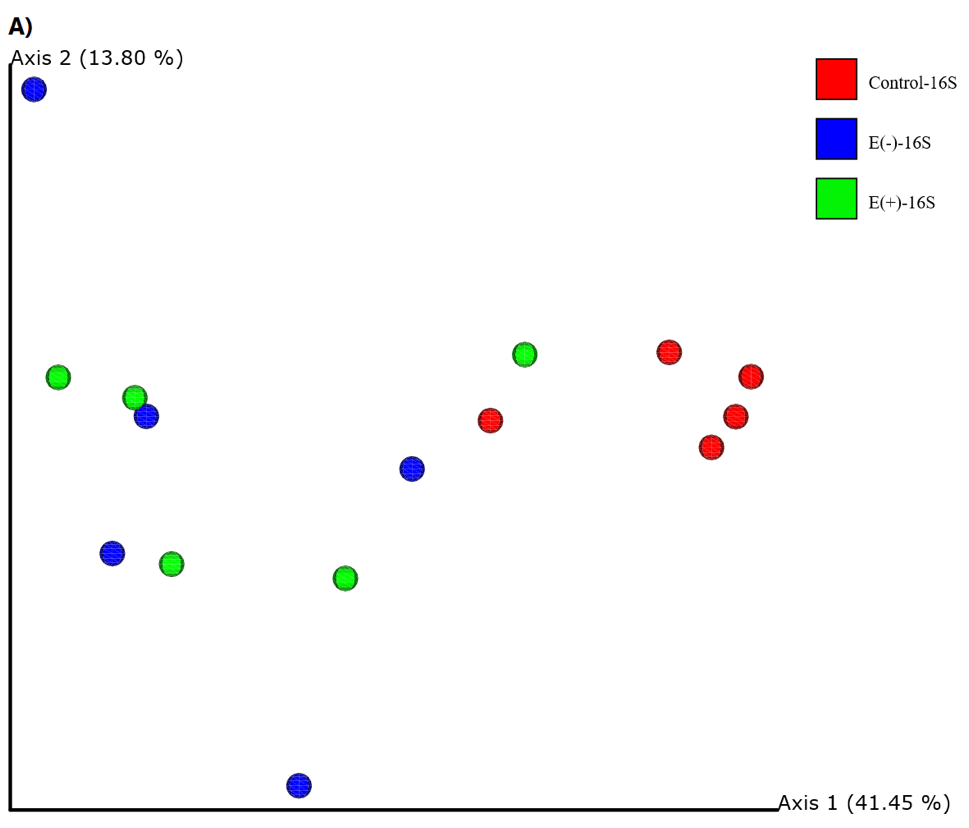


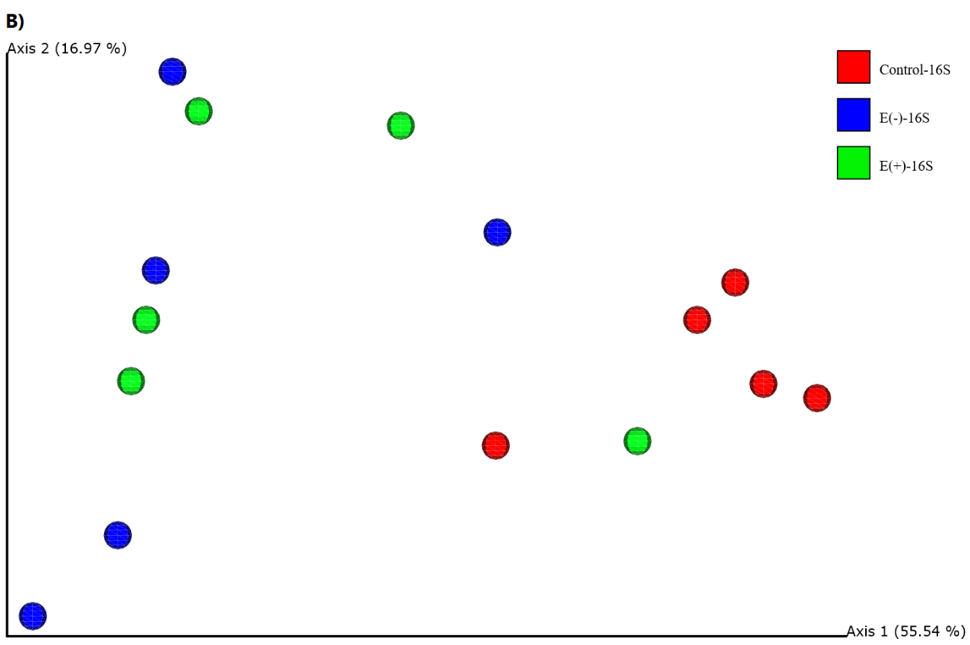

Supplement: Supplementary file 2 [file Data_Sheet_2.doc]
