# Supplementary material for: Symbiotic Interaction Enhances the Recovery of Endangered Tree Species in the Fragmented Maulino Forest
Source: Front Plant Sci. 2021 Apr 15;12:663017. doi: 10.3389/fpls.2021.663017 (PMC8081837; doi:10.3389/fpls.2021.663017)
Supplement: Supplementary file 3 [file Data_Sheet_3.doc]

**Suplemmentary material S3.** Effects of the inoculation with comercial mix of fugus, Antarctic endophytes and control (without inoculation) on the net photosynthetic rate (A max), water use efficiency (WUE), cumulative growth and survival percentage in plants of two endangered Maulino forest trees: *Nothofagus alessandrii* and *Nothofagus glauca*. Effect on Amax, WUE and cunulative growth were recorded at 3 months, while suvival percentaje was recorded at 6 months. Mean values and ±1SD are shown and different letters indicated significant difference by Tukey´s test.

| **Species** | **Variable** | **Control** | **Commercial**  **mycorrhizae** | **Antarctic**  **endophytes** |
| --- | --- | --- | --- | --- |
| ***N. alessandrii*** | **A max** | 8.4 ±0.3c | 9.9 ±0.3b | 12.7 ±0.6a |
|  | **WUE** | 2.4 ±0.2c | 3.1 ±0.2b | 3.9 ±0.3a |
|  | **Growth** | 1.8 ±0.2b | 2.4 ±0.3a | 2.9 ±0.3a |
|  | **Survival** | 20% | 35% | 55% |
| ***N. glauca*** | **A max** | 9.0 ±0.3c | 12.2 ±0.6b | 14.6 ±0.4a |
|  | **WUE** | 3.0 ±0.2b | 3.7 ±0.3a | 4.0 ±0.2a |
|  | **Growth** | 2.9 ±0.2c | 4.0 ±0.1b | 4.8 ±0.2a |
|  | **Survival** | 25% | 45% | 55% |
